# Complementary feeding and food‐group level inequality among Ethiopian children 6–23 months of age (2011–2019)

**DOI:** 10.1111/mcn.13375

**Published:** 2022-05-22

**Authors:** Woinshet Tizazu, Arnaud Laillou, Bayuh Asmamaw Hailu, Stanley Chitekwe, Kaleab Baye

**Affiliations:** ^1^ Center for Food Science and Nutrition Addis Ababa University Addis Ababa Ethiopia; ^2^ Nutrition Section, UNICEF Ethiopia Addis Ababa Ethiopia; ^3^ Monitoring and Evaluation Wollo University Dessie Ethiopia; ^4^ Research center for Inclusive Development in Africa (RIDA) Addis Ababa Ethiopia

**Keywords:** affordability, breastfeeding, complementary food, diet quality, food system, inequality

## Abstract

Ensuring diet quality in the first 2 years of life is critical to preventing malnutrition and instilling healthy food preferences. Children's diet quality has changed little over time and inequalities by socioeconomic status, rural–urban residence, but also by food group may exist. Using data from the 2011, 2016 and 2019 demographic and health surveys (DHS), we estimated the prevalence and inequalities in the minimum diet diversity (MDD), minimum meal frequency (MMF) and minimum acceptable diet (MAD). We further assessed food group‐level inequities. In 2019, only 13.5% of children 6–23 months of age met the MDD, 55% met the MMF and only 11% met the MAD indicator. Absolute and relative measures of inequality were calculated. Modest increases in MDD, MMF and MAD were observed over the past decade (2011–2019). These modest improvements were concentrated in limited geographical areas, among children in wealthier households, and urban residents. Unhealthy practices such as bottle‐feeding and zero fruit and vegetables have been increasing; whereas, inequities in the consumption of nutrient‐dense foods have widened. Nevertheless, children from the wealthiest quintile also failed to meet the MDD. Multisectoral efforts that span from diversifying the food supply, regulating the marketing of unhealthy foods, and promoting minimal processing of perishables (i.e., to extend shelf‐life) are needed. Context‐adapted behavioural change communication along with nutrition‐sensitive social protection schemes are also needed to equitably improve the diet quality of children in Ethiopia.

## Key messages

- Improvements in child diet quality are concentrated in limited geographical areas, primarily among wealthier households, and urban residents.
- Unhealthy practices such as bottle‐feeding and zero fruit and vegetables have been increasing over the past decade.
- Inequalities in the consumption of nutrient‐dense foods have been widening in the last decade.

## Graphical abstract

Improvements in child diet quality are concentrated in limited geographical areas, primarily among wealthier households and urban residents. In contrast, unhealthy practices such as bottle‐feeding and zero fruit and vegetables have been increasing over the past decade.

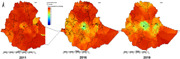

## 1 BACKGROUND

Despite significant progress over the last decade, child malnutrition remains a major public health concern in low and middle‐income countries (LMICs). Globally, in 2020, 149 million children under 5 years of age were stunted, 45 million wasted and 462 million adults were underweight (SOFI, 2021). Many countries, including Ethiopia, are off‐track from the Sustainable Development Goal (SDG) nutrition targets, and thus need to accelerate and intensify efforts to address child malnutrition (Laillou et al., 2020). The key to such efforts is to ensure that all children have nutrient‐adequate and healthy diets (Baye, 2021). However, a recent study looking at dietary diversity in 49 LMICs showed that only four countries had >50% of their children meeting the minimum dietary diversity (MDD)(Baye & Kennedy, 2020).

Children's diet quality has been consistently associated with income, maternal literacy, as well as the underpinning food supply and food environment (Baye & Kennedy, 2020). Although nutrition education interventions have been promoting dietary diversity, progress has been slow and unequal. The recent 'Fill the nutrient gap' report for Ethiopia revealed that a substantial proportion (>60%) of Ethiopian households cannot afford the minimum cost‐nutritious diet, modelled for a five‐member household that included nutritionally vulnerable groups like lactating women, adolescents and children under 2 years of age (WFP/EPHI, 2021). Consequently, adopting a nutritious diet for all members of the household is currently challenging and requires a major food systems transformation. However, in the meantime, ensuring diet quality for infants and young children in the first 2 years of life may be possible, given the very small portion size these children consume but require bold food systems measures to make nutritious foods available, accessible and affordable. Also given the rapid growth and the long‐term consequences that early undernutrition poses, ensuring that diets of children are nutritionally adequate should be a priority (Baye, 2021; Dewey & Begum, 2011).

The SDG calls for ending all forms of malnutrition (SDG 2), ensuring good health and well‐being for all (SDG3), and reducing inequalities (SDG 10), all part of the core guiding principle of the SDGs: ‘leaving no one behind’ and ensuring that basic human rights are upheld. Adopting these guiding principles, however, requires a better understanding of whether progress on diet quality‐ is made and how equitable these signs of progress are is central to ending all forms of malnutrition and to laying the foundation of good health (Baye, 2017). A few multi‐country studies have recently evaluated inequalities in diet quality indicators like MDD, but more detailed country‐level analyses including the recently developed indicators on unhealthy feeding practices are needed to support the Ethiopian food system policy and programmes (Baye & Kennedy, 2020; Baye et al., 2020; Gatica‐Domínguez et al., 2021). Besides, understanding food‐group level inequities can help shape food systems' related policies by identifying priorities and indicating which food groups may need subsidies.

In this study, we analysed data from the Ethiopian Demographic and Health Surveys (DHS 2011, 2016 and 2019) to present the spatial and temporal trends in diet quality indicators for children 6–23 months of age, disaggregated by wealth quintile and rural–urban residence. We used the most recent 2021 Infant and Young Child Feeding (IYCF) indicators which allowed us to assess not only proxies of energy and nutrient adequacy but also capture some aspects of unhealthy feeding practices. We then identified temporal and food group‐level inequalities to inform the design and implementation of more effective and inclusive food systems' programmes and policies.

## 2 METHODS

### 2.1 Overview and data source

We estimated and mapped the prevalence of children (6–23 months) that did not meet the MDD using the latest three rounds of the Ethiopian Demographic and Health Surveys (EDHS 2011–2019). Enumeration areas where dietary diversity indicators were taken were linked to the geographical coordinates using a global positioning system (GPS). Enumeration areas for DHS surveys are clusters from which households are sampled. The sub‐national prevalence of children not meeting the MDD was estimated and mapped. For unmeasured areas, the prevalence was predicted using ordinary Kriging. Inequalities in meeting the MDD by rural–urban and wealth quintiles were explored.

### 2.2 Outcomes

The outcomes of interest were the prevalence of children meeting the MDD, minimum meal frequency (MMF), minimum acceptable diet (MAD), zero‐fruit and vegetable consumption and bottle feeding as defined by the recently updated IYCF indicators (WHO, 2021). The MDD was met when at least five of the eight food groups were reported to be consumed in the last 24 h. The eight food groups used to calculate the MDD were: (i) breastmilk; (ii) grains, roots and tubers; (iii) legumes and nuts; (iv) dairy; (v) flesh foods (meat, fish and poultry); (vi) eggs; (vii) vitamin A‐rich fruits and vegetables; and (viii) other fruits and vegetables.

MMF was calculated as the number of breastfed children who consumed solid, semisolid or soft foods at least twice (for age 6–8 months) or three times (for age 9–23 months), plus the number of nonbreastfed children who received ≥4 feeds, during the previous day (including ≥ 1 feed of solid, semisolid or soft foods); the resulting sum is divided by the number of children aged 6–23 months.

The MAD was calculated as the percentage of children who meet both the MDD and MMF, and who were either breastfed or had ≥2 nonhuman milk feeds in the previous 24 h.

We also estimated the prevalence of unhealthy feeding practices like bottle‐feeding and zero fruit and vegetable consumption.

### 2.3 Inequality measures

We disaggregated MDD, MMF and MAD indicators by wealth quintile and rural/urban residence. Urban and rural residence was classified according to boundaries provided by local authorities. The wealth index was derived from principal component analyses applied to a list of household assets/characteristics, which are country‐specific. The first quintile (Q1) represents the 20% poorest families, and the last quintile (Q5) represents the 20% wealthiest families. Quintiles correspond to the relative position of households within each national sample, rather than absolute income for which data are not available for most studies. As fertility is higher among the households in low socioeconomic status (SES), the lowest wealth quintile tends to include >20% of all children surveyed, whereas the highest wealth quintiles include <20% of all children. The food groups consumed over the last 24 h were presented.

We estimated the absolute and relative inequalities by calculating the percentage point difference between the wealthiest (Q5) and poorest quintiles (Q1), and the ratio between Q5 and Q1 (Q5/Q1) to estimate relative gaps between extremes in SES. We then calculated the slope index of inequality (SII) and the concentration index (CIX), which both account for the entire SES distribution (Q1–Q5). The SII is expressed in percentage points and CIX as a range between −1 and +1. For both SII and CIX, a value of zero represents absolute equality between the rich and the poor, positive values indicate a pro‐rich distribution, and negative values indicate a pro‐poor distribution. The CIX values are then multiplied by 100 for presentation. The SII was estimated by using a regression approach, and the CIX was calculated using an analogous approach by ranking individuals according to SES position. These two measures were also used to assess whether inequalities increased or declined over time.

### 2.4 Statistical analysis

We estimated the prevalence of MDD, MMF and MAD along with their 95% confidence intervals for the years 2011, 2016 and 2019. We presented these IYCF indicators by wealth quintile and rural–urban residence. Equiplots were used to depict inequalities by wealth and rural/urban residence. We mapped the prevalence of children who do not meet the MDD and forecasted the prevalence in unmeasured areas using spatial interpolation. For spatial interpolation, we used the ordinary kriging method using system for automated geoscientific analyses‐geographic information system. Statistical analyses were conducted using Stata v14. Factors associated with MDD were identified using multilevel logistic regression. A mixed‐effect logistic regression model was run, and adjusted odds ratios (AORs) with corresponding 95% confidence intervals were estimated.

## 3 RESULTS

**Figure 1:**
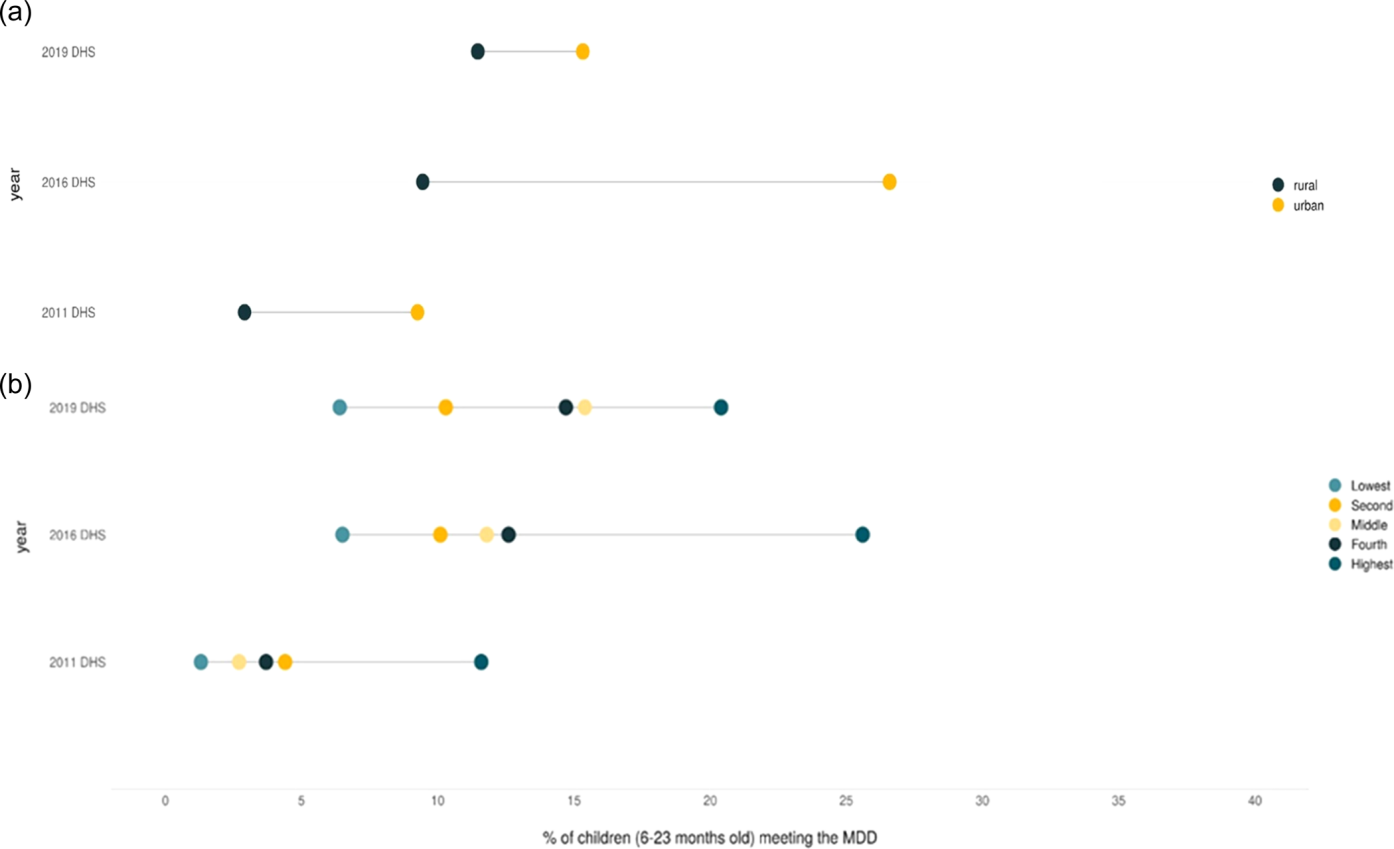
Trend and inequalities in child diet quality (MDD) indicators by rural–urban and wealth quintile, 2011, 2016 and 2019.

Modest increases in MDD, MMF and MAD were observed over the past decade (2011–2019). In 2019, only 13.5% of children 6–23 months of age met the MDD (Table 1). About 55% of the children met the MMF. Altogether, only 11% met the composite MAD indicator**.** Figure 1 presents equiplots presenting the proportion of children who met the MDD in 2011, 2016 and 2019 by rural–urban residence. Rural areas have seen consistent increases in the proportion of children who met the MDD. About 13.5% of the children met the MDD in 2019, compared with only 3.5% in 2011. Rural areas were always behind their urban counterparts. The rural–urban inequality was the highest in 2016 and the least in 2019, suggesting that urban–rural gaps are reducing, partly because the proportion of children who met the MDD in urban areas decreased between 2016 and 2019.

**Table 1:** Inequalities in diet quality indicators, 2019

	Prevalence [95% confidence interval]			Absolute and relative inequality measures					
	National	Q1	Q5	Q5‐Q1	Q5/Q1	SII	*p* value	CIX	*p* value
IYCF indicators									
MDD	13.5 [10.5,17.3]	6.4 [3.1,12.7]	20.4 [13.6,29.5]	14	3.2	16.6 [6.0, 27.1]	0.002	19.9 [7.2, 32.6]	0.002
MMF	55.5 [50.7,60.2]	36.7 [29.0,45.0]	70.1 [59.4,78.9]	33.4	1.9	37.9 [23.4, 52.3]	<0.001	11.1 [6.9, 15.3]	<0.001
MAD	11.3 [8.6,14.7]	2.6 [0.9,7.4]	17.4 [11.4,25.7]	14.8	6.7	16.7 [7.7, 25.7]	<0.001	24.1 [11.2, 37.0]	<0.001
Food groups consumed									
Breastmilk	85.3 [82.0,88.0]	76.3 [69.7,81.9]	80 [68.9,87.9]	3.7	1.0	2.9 [−9.0, 14.7]	0.633	0.6 [−1.6, 2.8]	0.568
Grains, roots, tubers	71.3 [67.9,74.5]	64.1 [56.5,71.0]	81 [70.6,88.3]	16.9	1.3	15.6 [3.2, 28.0]	0.014	3.4 [0.6, 6.2]	0.018
Dairy products	34.9 [30.5,39.6]	32 [24.1,41.0]	43.9 [33.1,55.4]	11.9	1.4	14.4 [−1.6, 30.5]	0.077	6.6 [−0.9, 14.1]	0.083
Legumes and nuts	24.9 [21.4,28.7]	18.2 [12.7,25.3]	28.8 [21.6,37.2]	10.6	1.6	10.9 [−0.6, 22.5]	0.064	7.1 [−0.4, 14.6]	0.064
Eggs	18.2 [14.9,22.0]	9.3 [5.6,14.9]	24.2 [16.1,34.6]	14.9	2.6	15.4 [2.9, 28.0]	0.016	13.7 [2.4, 24.9]	0.017
Vitamin A‐rich fruits and vegetables	26.7 [23.0,30.9]	10.8 [6.5,17.5]	36.5 [26.7,47.4]	25.7	3.4	28.2 [14.7, 41.7]	<0.001	17.2 [9.0, 25.3]	<0.001
Other fruits and vegetables	10.7 [8.2,13.8]	3.3 [1.4,7.4]	17.7 [11.4,26.3]	14.4	5.4	17 [7.5, 26.5]	0.001	25.8 [11.4, 40.1]	<0.001
Flesh: meat, poultry, fish	8.8 [5.9,13.0]	3.4 [1.5,7.2]	17.4 [11.9,24.7]	14	4.1	17.5 [9.8, 25.2]	<0.001	32.3 [18.2, 46.3]	<0.001
Unhealthy feeding practice									
Zero fruits and variables	69.3 [64.6,73.7]	87.3 [80.6,91.9]	57.5 [45.1,69.0]	29.8	0.7	33.6 [49.4, 17.9]	<0.001	−7.9 [−11.6, −4.2]	<0.001
Bottle‐feeding	25.4 [21.6,29.6]	18.8 [11.3,29.5]	41.1 [34.2,48.4]	22.3	2.2	31.2 [16.9, 45.6]	<0.001	20.1 [10.8, 29.4]	<0.001

*Note*: CIX was multiplied by 100 for presentation purposes; *p*‐values represent significant difference by wealth quintile; values are point estimates (means) with their 95% confidence intervals in brackets.

Abbreviations: CIX, concentration index; IYCF, infant and young child feeding; Q1, quintile 1, Q5, quintile 5; SII, slope index inequality.

The proportion of children that met the MDD by wealth quintile showed wide variations between the richest and the poorest. Nevertheless, even in the highest wealth quintile, less than 30% of children met the MDD. The poorest wealth quintiles witnessed some progress between 2011 and 2016, but no improvements were observed between 2016 and 2019. Consequently, the MDD prevalence remained below 10%.

**Figure 2:**
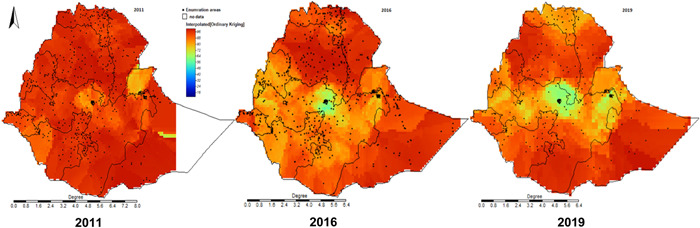
Subnational spatial and temporal trends in the prevalence of children meeting the minimum dietary diversity.

Figure 2 presents the geospatial trend and distribution in the prevalence of children that met the MDD. In 2011, almost all of the country had a very high proportion of children not meeting the MDD. In 2016, and later in 2019, pockets of improvements have been observed in central Ethiopia, surrounding the capital city, and areas in the northern and eastern parts of the country. Little progress was observed in Amhara, Afar, Southern Oromia and Somali regions.

**Figure 3:**
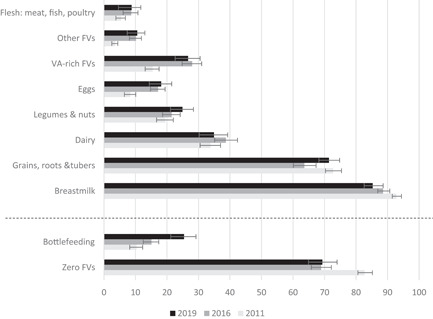
Food groups consumed by children 6–23 months of age, 2011, 2016 and 2019.

Nutrient‐dense food groups like animal source foods (ASF) and eggs were consumed by less than one‐fifth of the children (Figure 3). Although consumption of fruits and vegetables and eggs consumption has shown slight increases between 2011 and 2016, it remains low. No significant changes were observed between 2016 and 2019. Similarly, the proportion of children with zero fruits and vegetables decreased from 93% in 2011 to 69% in 2016 but showed no change after then (Supporting Information Material). Breastfeeding rates were high, but showing a declining trend over the years, even though it is still above 80%; whereas bottle‐feeding has been significantly increasing over the past decade (2011–2019).

**Figure 4:**
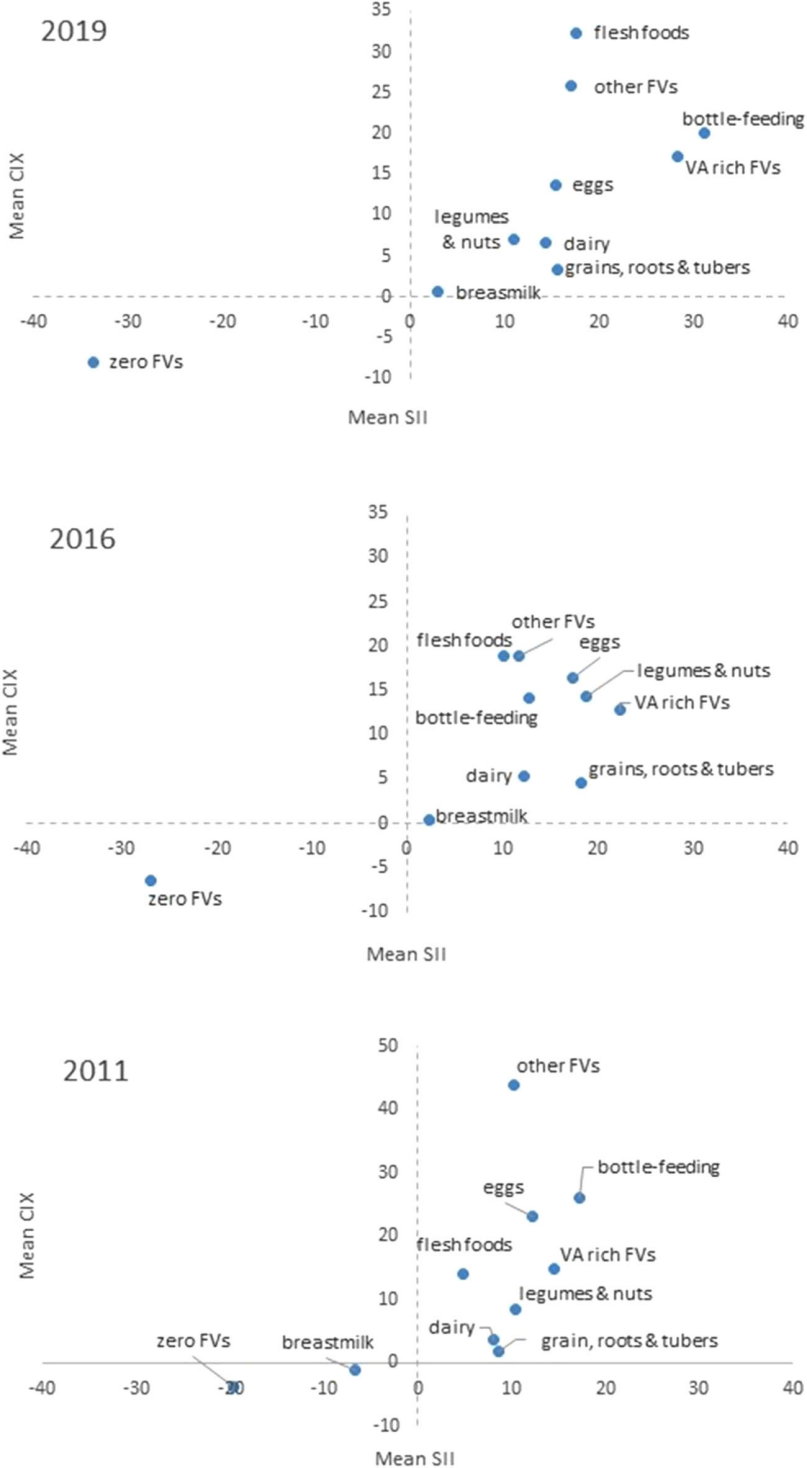
Food group‐level inequality, 2011–2019. SII reflects absolute inequality; whereas CIX reflects relative inequality between the highest and the lowest wealth quintile; positive values present a pro‐rich distribution, whereas negative values represent a pro‐poor distribution; the origin of the graph (0,0) represents absolute equality. Values further away from zero represent higher inequality. CIX, concentration index.

In 2019, almost all food groups showed pro‐rich distribution (Figure 4), except for the unhealthy practice of zero fruit and vegetables which showed pro‐poor distribution. The highest inequality, illustrated by points further away from zero (equality), was observed for flesh foods, eggs and fruits and vegetables (*p* < 0.05). The least inequality was observed for breastmilk, but pro‐rich distribution was observed for bottle‐feeding. The prevalence of MDD among the richest wealth quintile (Q5) was three times higher than in the poorest wealth quintile (Table 1). The consumption of nutrient‐dense foods like eggs (2.6×) and ASF (4.1×) is higher in the richest (Q5) compared with the poorest quintile (Q1).

## 4 DISCUSSION

This study showed that despite some improvements, the diet quality of children 6–23 months of age has remained unacceptably low. The modest improvements observed were concentrated in limited geographical areas, primarily among children in wealthier households, and urban residents. Unhealthy practices such as bottle‐feeding and zero fruit and vegetables have been increasing over the past decade. Unhealthy food practices were more prevalent than recommended IYCF practices. Inequalities in the consumption of nutrient‐dense foods like fruits and vegetables, and ASF are present and have been widening in the last decade.

Although breastfeeding was highly prevalent and was the most equitably distributed practice, the rise in bottle‐feeding raises several concerns. First, such practice has been linked to poor oral health and increases the risk of overweight/obesity (Brown et al., 1989; Li et al., 2012); second, the increase in bottle feeding can imply the increase in the use of breastmilk substitutes but can also negatively affect suckling behaviour (WHO, 2005); and finally, if bottles are not properly washed, they can expose children to gastrointestinal infections, which can further compromise the health, growth and wellbeing of children (Lutter et al., 2021). Indeed, a recent study has shown that the monitoring and regulation of the marketing of follow‐up formulas and complementary foods in Ethiopia are suboptimal (Laillou et al., 2021). This along with the poor quality complementary diets can trap children into a vicious cycle of infection and poor appetite, possibly explaining the high prevalence of malnutrition (Abebe et al., 2017). To counter the increasingly aggressive promotion of breastmilk substitutes (BMS), revisions and enforcement of the national laws and regulations in line with international codes of marketing of BMS are needed (Laillou et al., 2021; Michaud‐Létourneau et al., 2019).

The alarmingly low MDD prevalence along with the very slow progress observed over the last decade is a matter of concern. A diet lacking diversity has been associated with poor child growth (Baye & Kennedy, 2020; Baye et al., 2020), micronutrient deficiencies (Moursi et al., 2008) and eating disorders like picky eating (Taylor & Emmett, 2019; Taylor et al., 2015). Such negative outcomes experienced during early childhood are also likely to lead to serious cognitive and developmental delays, with possible long‐term consequences (Dewey & Begum, 2011; Prado & Dewey, 2014). Indeed, undernutrition in early life has been associated with poor productivity and health outcomes in later life (Hoddinott et al., 2008). Consequently, inequalities in diet quality in early life are likely to lead to wider inequalities in health and wellbeing. More pronounced and growing inequalities have been observed for nutrient‐dense foods like flesh foods, eggs, fruits and vegetables. These nutrient‐dense foods showed pro‐rich distribution, whereas the unhealthy practice of zero fruit and vegetables showed pro‐poor distribution. This is in line with a recent global analysis of inequalities in diet diversity in 80 LMICs (Gatica‐Domínguez et al., 2021). Zero fruit and vegetable among infants and young children is considered an indicator of unhealthy practice in the 2021 IYCF indicators (WHO, 2021), because such behaviour is believed to track over time, leading to low fruit and vegetable consumption in adulthood (Nicklaus & Remy, 2013; Nicklaus et al., 2004), a leading risk factor for cardiovascular diseases.

The unaffordability of nutrient‐dense foods has been a growing area of concern impeding the adoption of nutritious diets. Recent studies from LMICs have shown that the price of nutrient‐dense foods is exorbitantly higher than that of starchy staples (Headey & Alderman, 2019; Masters et al., 2018). A global analysis comprising 177 countries has shown that a nutrient adequate diet costs 2.66 times the cost of subsistence daily energy, and this figure was much higher in Sub‐Saharan Africa (Bai et al., 2021). In Ethiopia, the price of nutrient‐dense foods has increased significantly over the last decade (2011–2017), while during the same period the price of sugar, honey, oil and starchy staples remained stable or even showed some declines in price (Baye & Hirvonen, 2020). Such price changes can thus further discourage the adoption of a more diversified diet, which partly explains the reduced prevalence of MDD in 2019 seen among the urban and the wealthiest quintile.

Most nutrient‐dense foods are perishable, leading to losses and food safety concerns in the absence of proper storage (i.e., refrigeration) or processing that extends shelf‐life (e.g., drying) (Kechero et al., 2019). The lack of electricity and refrigeration in most parts of rural Ethiopia has thus been limiting the access and consumption of even the cheapest forms of fruits and vegetables (e.g., pumpkin). Indeed, a recent study has shown that the affordability of a nutritious diet is negatively associated with rural travel times and access to electricity (Bai et al., 2021). This implies that food transformation in a form of minimal processing that helps prevent postharvest loss, increase income and extend shelf‐life are direly needed to facilitate the storage, transportation and distribution of nutrient‐dense foods in such settings (Monteiro et al., 2021). This potential has been illustrated by recent studies from Ethiopia that reported on the dehydration of fruits and eggs to increase their availability, accessibility and affordability (Abreha et al., 2021; Minuye et al., 2021).

Clearly increasing incomes and empowering women is critical to improving the diet quality of infants and young children (Bai et al., 2021; Baye et al., 2021). However, the low MDD prevalence, even among the highest wealth quintile, suggests that factors other than income and affordability are important (Baye, 2021). Indeed, using income elasticities to measure how consumption is expected to vary with changes in real incomes, a recent study showed that cereals are favoured in rural areas; whereas, meat and fruits are favoured in urban areas. Cultural and religious practices such as the extended fasting periods during which devout Orthodox Christians abstain from ASFs are also key drivers shaping food consumption (Hirvonen et al., 2016). The food supply is also predominantly cereal‐based with limited production of fruit and vegetables, which can in turn affect availability, accessibility and affordability (Baye et al., 2019). Altogether, this suggests that context‐adapted behavioural change communication interventions are needed, including among adolescents and youth. To be effective, such interventions should be cognizant of the structural, physical (e.g. agroecology), social and behavioural drivers that encourage or discourage the consumption of specific foods.

This study has several limitations that need to be considered. First, although we used three rounds of the Ethiopian DHS, allowing us to observe temporal trends in diet quality, the cross‐sectional nature of the surveys does not allow us to make causal inferences. Second, unlike the 2011 and 2016 rounds, the 2019 data set is from a mini‐DHS. Third, the DHS has limited indicators related to unhealthy feeding practices; consequently, only data related to bottle‐feeding and zero fruit and vegetable consumption is presented for this dimension.

Notwithstanding the above limitations, this study has shown that infants and young children in Ethiopia have diets that lack diversity. Inequalities between rural–urban residence and wealth quintile are stark. The highest inequalities are observed for nutrient‐dense foods like ASF, fruits, and vegetables. Nevertheless, even among children in the wealthiest quintiles, a substantial proportion of children failed to meet the MDD, MMF and MAD. Bold and multi‐sectoral efforts are needed to improve the diet quality of children by promoting healthy consumption and discouraging unhealthy feeding practices. This could require diversifying the food supply, increasing incomes, promoting minimal processing that can extend the shelf‐life of perishable foods, shorter value‐chains, designing and implementing effective context‐adapted behaviour change communication, as well as bridging inequalities through subsidies and social protection schemes that ensure that no child is left behind. The level of coordination, partnership and programme implementation along these and other sectors will determine our success in meeting commitments to a prosperous, equitable, and healthier future for today's children.

## AUTHOR CONTRIBUTIONS

Woinshet Tizazu, Kaleab Baye, Stanley Chitekwe, and Arnaud Laillou conceived the study. Woinshet Tizazu, Bayuh Assamnew, and Kaleab Baye prepared and analysed the data; Kaleab Baye and Woinshet Tizazu wrote the paper with inputs from Arnaud Laillou, Stanley Chitekwe, and Bayuh Assamnew. All authors read and approved the final manuscript.

## ACKNOWLEDGEMENTS

This study was funded by UNICEF Ethiopia.

## CONFLICTS OF INTEREST

The authors declare no conflicts of interest.

## Supporting information

Supporting information.

## Data Availability

The data that support the findings of this study are available from the demographic and health survey database https://dhsprogram.com/after registration.

## References

[mcn13375-bib-0001] Abebe, Z. , Haki, G. D. , & Baye, K. (2017). Child feeding style is associated with food intake and linear growth in rural Ethiopia. Appetite, 116, 132–138. 10.1016/j.appet.2017.04.033 28461197

[mcn13375-bib-0002] Abreha, E. , Getachew, P. , Laillou, A. , Chitekwe, S. , & Baye, K. (2021). Physico‐chemical and functionality of air and spray dried egg powder: Implications to improving diets. International Journal of Food Properties, 24(1), 152–162. 10.1080/10942912.2020.1867569

[mcn13375-bib-0003] Bai, Y. , Alemu, R. , Block, S. A. , Headey, D. , & Masters, W. A. (2021). Cost and affordability of nutritious diets at retail prices: Evidence from 177 countries. Food Policy, 99, 101983. 33767525 10.1016/j.foodpol.2020.101983 PMC7970354

[mcn13375-bib-0004] Baye, K. (2017). The Sustainable Development Goals cannot be achieved without improving maternal and child nutrition. Journal of Public Health Policy, 38(1), 137–145. 10.1057/s41271-016-0043-y 28275250

[mcn13375-bib-0005] Baye, K. (2021). Improved diet quality, a missing ingredient for accelerating stunting reduction: An example from Ethiopia. Archives of Disease in Childhood, 107, 5–6. 33402327 10.1136/archdischild-2020-320292

[mcn13375-bib-0006] Baye, K. , & Hirvonen, K. (2020). Accelerating progress in improving diets and nutrition in Ethiopia (144). Intl Food Policy Res Inst.

[mcn13375-bib-0007] Baye, K. , Hirvonen, K. , Dereje, M. , & Remans, R. (2019). Energy and nutrient production in Ethiopia, 2011‐2015: Implications to supporting healthy diets and food systems. PLoS One, 14(3):e0213182. 10.1371/journal.pone.0213182 30861012 PMC6413914

[mcn13375-bib-0008] Baye, K. , & Kennedy, G. (2020). Estimates of dietary quality in infants and young children (6–23 mo): Evidence from demographic and health surveys of 49 low‐and middle‐income countries. Nutrition, 78, 110875. 32653760 10.1016/j.nut.2020.110875

[mcn13375-bib-0009] Baye, K. , Laillou, A. , & Chitweke, S. (2020). Socio‐economic inequalities in child stunting reduction in sub‐Saharan Africa. Nutrients, 12(1):253. 10.3390/nu12010253 31963768 PMC7019538

[mcn13375-bib-0039] Baye, K. , Laillou, A. , & Chitweke, S. (2021). Empowering women can improve child dietary diversity in Ethiopia. Maternal & Child Nutrition, 20(S5), e13285. 10.1111/mcn.13285 PMC11258775 34738293

[mcn13375-bib-0010] Brown, K. H. , Black, R. E. , de Romaña, G. L. , & de Kanashiro, H. C. (1989). Infant‐feeding practices and their relationship with diarrheal and other diseases in Huascar (Lima), Peru. Pediatrics, 83(1), 31–40. 2909974

[mcn13375-bib-0011] Dewey, K. G. , & Begum, K. (2011). Long‐term consequences of stunting in early life. Maternal & Child Nutrition, 7, 5–18. 21929633 10.1111/j.1740-8709.2011.00349.x PMC6860846

[mcn13375-bib-0012] Gatica‐Domínguez, G. , Neves, P. A. R. , Barros, A. J. D. , & Victora, C. G. (2021). Complementary feeding practices in 80 low‐and middle‐income countries: Prevalence of and socioeconomic inequalities in dietary diversity, meal frequency, and dietary adequacy. The Journal of Nutrition, 151(7), 1956–1964. 33847352 10.1093/jn/nxab088 PMC8245881

[mcn13375-bib-0013] Headey, D. D. , & Alderman, H. H. (2019). The relative caloric prices of healthy and unhealthy foods differ systematically across income levels and continents. The Journal of Nutrition, 149(11), 2020–2033. 31332436 10.1093/jn/nxz158 PMC6825829

[mcn13375-bib-0016] Hirvonen, K. , Taffesse, A. S. , & Hassen, I. W. (2016). Seasonality and household diets in Ethiopia. Public Health Nutrition, 19(10), 1723–1730. 26585676 10.1017/S1368980015003237 PMC10271090

[mcn13375-bib-0017] Hoddinott, J. , Maluccio, J. A. , Behrman, J. R. , Flores, R. , & Martorell, R. (2008). Effect of a nutrition intervention during early childhood on economic productivity in Guatemalan adults. The Lancet, 371(9610), 411–416. 10.1016/S0140-6736(08)60205-6 18242415

[mcn13375-bib-0018] Kechero, F. K. , Baye, K. , Tefera, A. T. , & Tessema, T. S. (2019). Bacteriological quality of commonly consumed fruit juices and vegetable salads sold in some fruit juice houses in Addis Ababa, Ethiopia. Journal of Food Safety, 39(1), e12563. 10.1111/jfs.12563

[mcn13375-bib-0019] Laillou, A. , Baye, K. , Meseret, Z. , Darsene, H. , Rashid, A. , & Chitekwe, S. (2020). Wasted children and wasted time: A challenge to meeting the nutrition sustainable development goals with a high economic impact to Ethiopia. Nutrients, 12(12), 3698. 33266008 10.3390/nu12123698 PMC7760409

[mcn13375-bib-0020] Laillou, A. , Gerba, H. , Zelalem, M. , Moges, D. , Abera, W. , Chuko, T. , Getahun, B. , Kahsay, H. , & Chitekwe, S. (2021). Is the legal framework by itself enough for successful WHO code implementation? A case study from Ethiopia. Maternal & Child Nutrition, 17(1), e13059. 32841521 10.1111/mcn.13059 PMC7729794

[mcn13375-bib-0021] Li, R. , Magadia, J. , Fein, S. B. , & Grummer‐Strawn, L. M. (2012). Risk of bottle‐feeding for rapid weight gain during the first year of life. Archives of Pediatrics & Adolescent Medicine, 166(5), 431–436. 22566543 10.1001/archpediatrics.2011.1665

[mcn13375-bib-0022] Lutter, C. K. , Grummer‐Strawn, L. , & Rogers, L. (2021). Complementary feeding of infants and young children 6 to 23 months of age. Nutrition Reviews, 79, 825–846. 33684940 10.1093/nutrit/nuaa143

[mcn13375-bib-0023] Masters, W. A. , Bai, Y. , Herforth, A. , Sarpong, D. B. , Mishili, F. , Kinabo, J. , & Coates, J. C. (2018). Measuring the affordability of nutritious diets in Africa: price indexes for diet diversity and the cost of nutrient adequacy. Wiley Online Library. 10.1093/ajae/aay059 PMC7053386 32139915

[mcn13375-bib-0024] Michaud‐Létourneau, I. , Gayard, M. , & Pelletier, D. L. (2019). Translating the international code of marketing of breast‐milk substitutes into national measures in nine countries. Maternal & Child Nutrition, 15, e12730. 30793543 10.1111/mcn.12730 PMC6519018

[mcn13375-bib-0025] Minuye, M. , Getachew, P. , Laillou, A. , Chitekwe, S. , & Baye, K. (2021). Effects of different drying methods and ascorbic acid pretreatment on carotenoids and polyphenols of papaya fruit in Ethiopia. Food Science & Nutrition, 9(6), 3346–3353. 34136199 10.1002/fsn3.2324 PMC8194739

[mcn13375-bib-0026] Monteiro, C. A. , Lawrence, M. , Millett, C. , Nestle, M. , Popkin, B. M. , Scrinis, G. , & Swinburn, B. (2021). The need to reshape global food processing: a call to the United Nations Food Systems Summit. BMJ Global Health, 6(7), e006885. 10.1136/bmjgh-2021-006885 PMC8319974 34321237

[mcn13375-bib-0027] Moursi, M. M. , Arimond, M. , Dewey, K. G. , Treche, S. , Ruel, M. T. , & Delpeuch, F. (2008). Dietary diversity is a good predictor of the micronutrient density of the diet of 6‐to 23‐month‐old children in Madagascar. The Journal of Nutrition, 138(12), 2448–2453. 19022971 10.3945/jn.108.093971

[mcn13375-bib-0028] Nicklaus, S. , Boggio, V. , Chabanet, C. , & Issanchou, S. (2004). A prospective study of food preferences in childhood. Food Quality and Preference, 15(7–8), 805–818.

[mcn13375-bib-0029] Nicklaus, S. , & Remy, E. (2013). Early origins of overeating: Tracking between early food habits and later eating patterns. Current Obesity Reports, 2(2), 179–184.

[mcn13375-bib-0030] Prado, E. L. , & Dewey, K. G. (2014). Nutrition and brain development in early life. Nutrition Reviews, 72(4), 267–284. 24684384 10.1111/nure.12102

[mcn13375-bib-0031] SOFI . (2021). The state of food security and nutrition in the world. Accessed August 20, 2021. 10.4060/cb4474en

[mcn13375-bib-0032] Taylor, C. M. , & Emmett, P. M. (2019). Picky eating in children: Causes and consequences. Proceedings of the Nutrition Society, 78(2), 161–169. 30392488 10.1017/S0029665118002586 PMC6398579

[mcn13375-bib-0033] Taylor, C. M. , Wernimont, S. M. , Northstone, K. , & Emmett, P. M. (2015). Picky/fussy eating in children: Review of definitions, assessment, prevalence and dietary intakes. Appetite, 95, 349–359. 26232139 10.1016/j.appet.2015.07.026

[mcn13375-bib-0034] WFP/EPHI . (2021). Fill the nutrient gap‐Ethiopia. Accessed August 15, 2021. https://www.wfp.org/publications/fill-nutrient-gap-ethiopia-summary-report-english

[mcn13375-bib-0035] WHO . (2005). WHO guiding principles of feeding non‐breastfed children 6‐24 months.

[mcn13375-bib-0036] WHO . (2021). *Indicators for assessing infant and young child feeding practices: Definitions and measurement methods.* World Health Organization and the United Nations Children's Fund (UNICEF).

