# Supplementary material for: Complementary feeding and food‐group level inequality among Ethiopian children 6–23 months of age (2011–2019)
Source: Matern Child Nutr. 2022 May 22;20(Suppl 5):e13375. doi: 10.1111/mcn.13375 (PMC11258773; doi:10.1111/mcn.13375)
Supplement: Supplementary file 1 — Supporting information. [file MCN-20-e13375-s001.docx]

**Fig S1** change in slope index inequality by year

MDD, minimum dietary diversity; MMF, minimum meal frequency; MAD, minimum adequate diet
